# Prescription patterns of analgesics in the last 3 months of life: a retrospective analysis of 10202 lung cancer patients

**DOI:** 10.1038/bjc.2011.150

**Published:** 2011-05-03

**Authors:** W Gao, M Gulliford, I J Higginson

**Affiliations:** 1 King's College London, School of Medicine, Department of Palliative Care, Policy and Rehabilitation, Cicely Saunders Institute, Bessemer Road, London SE5 9PJ, UK; 2 King's College London, Department of Primary Care and Public Health Sciences, Capital House, 42 Weston Street, London SE1 3QD, UK

**Keywords:** lung cancer, end of life, pain, analgesics, palliative care, prescription

## Abstract

**Background:**

To describe the prescription patterns of analgesics during the last 3 months of life in lung cancer and to determine the associated factors.

**Methods:**

Data on lung cancer patients (*N*=10 202) who died during 2000–2008 were extracted from the General Practice Research Database (GPRD). This database records prescriptions of patients received from UK general practices (GP), but not those from non-GP routes. Prescription prevalences were estimated. The associated factors were investigated using log-binomial regression.

**Results:**

The overall prescription prevalences were 50.4% (95% confidence interval (CI): 49.4–51.4%) for level 1 (e.g., paracetamol), 34.1% (95% CI: 33.2–35.0%) for level 2 (weak opioids), and 55.5 % (95% CI: 54.5–56.4%) for level 3 analgesics (strong opioids). Prescription prevalence of analgesics of all levels showed an increasing trend over the period 2000–2008 (annual increases range: 1.1–1.5%) but a decreasing trend with age (average decrease per group range: −5.8 to −1.8%). Patients in the older age groups were less likely to be prescribed level 3 analgesics than those in the younger age groups (PR_‘90+’ *vs* ‘<50’_=0.55 (95% CI: 0.45–0.67); PR_‘80−89’ *vs* ‘<50’_=0.73 (95% CI: 0.66–0.79); PR_‘70−79’ *vs* ‘<50’_=0.84 (95% CI: 0.77–0.90)).

**Conclusion:**

Analgesics have been increasingly prescribed in lung cancer. However, analgesics, especially at level 3, were relatively under-prescribed to people older than 70 years, warranting further investigation.

Lung cancer is the leading cause of death from cancer, responsible for over 1.4 million deaths worldwide and around 30 000 deaths in the United Kingdom each year (ONS, 2009; WHO, 2009). The worldwide projected deaths from lung cancer will rise up to nearly 2.3 million in 2030 (WHO, 2009). Over 70% of lung cancer patients die within a year after diagnosis (Rachet *et al*, 2009), leaving short time for care planning. Pain is one of the most common and most feared symptoms experienced by lung cancer patients. It affects 27% of outpatients and 76% of patients cared for by palliative care services, and significantly reduces quality of life (Di *et al*, 2004; Potter and Higginson, 2004; Costantini *et al*, 2009). Its prevalence increases in advanced disease. As death approaches, pain intensity increases (Morris *et al*, 1986). Therefore, good control of pain has been regarded as one of the primary goals of cancer care (Riley and Ross, 2005).

Pain may be related to the presence of primary or metastatic disease; it may also develop because of treatment. An understanding of the mechanism of the pain may be helpful in planning for optimal therapy (Levy, 1996; Bruera and Kim, 2003). The majority (80%) of cancer pains can be controlled with simple treatment. For the more complicated cases (20%), it is important to use a multidimensional approach that includes a careful re-assessment of the pain syndrome and use of second line agents and/or non-pharmacological interventions (Zech *et al*, 1995). For example, anti-hyperalgesic medications need to be considered earlier on in the prescribing process for the neuropathic pain (Ripamonti and Dickerson, 2001).

A three-step analgesic ladder has been proposed by the World Health Organization (WHO) for managing cancer pain (WHO, 1996). It includes five core principles for the management of pain: by the mouth (i.e., simple rather than invasive routes), by the clock (i.e., regularly rather than only as required), for the individual, with attention to detail and by the ladder (Jadad and Browman, 1995). This five-core principle is a stepwise approach to pain management. The first step involves using non-opioids analgesics for mild-to-moderate pain. Patients who fail the first-step regimen or who present with moderate-to-severe pain should be treated with weak opioids (step 2). For severe pain that cannot be controlled by step 2, treatment should proceed to stronger opioids (step 3). A systematic review found that 69–100% of patients received adequate analgesia when the guidelines were applied (Jadad and Browman, 1995).

However, recent surveys and meta-analysis of the available empirical evidence show that the undertreatment of cancer pain remains a widespread problem (Di *et al*, 2004; Deandrea *et al*, 2008). The inadequacy of analgesic treatment is even more apparent in the advanced stage of disease. An Italian survey conducted in patients with advanced non-small-cell lung cancer revealed that as high as 82% of those who reported pain received inadequate relief (Di *et al*, 2004).

Effective management of symptoms, including pain by primary care practitioners, is the key to providing quality end of life care and to enable more people approaching the end of their life to live and die in their preferred place, usually at home (Gomes and Higginson, 2006). The UK General Practice Research Database (GPRD) is the world's largest primary care database. The database holds full records for over 4 million currently registered patients and ∼11 million patients in total. These data are collected from around 520 primary care practices throughout the United Kingdom and cover 7% of the UK population. It contains patient-level longitudinal medical records in primary care and has the capacity to link to the other national databases. It has been widely used for a variety of research areas including pharmacoepidemiology, health service planning, and treatment patterns (GPRD, 2010).

The objectives of this study are to determine the prescription patterns of analgesics in the last year of life among patients with lung cancer, and factors associated with these patterns.

## Materials and methods

### Design

This is a population-based observational study.

### Data sources

The data for this study were extracted from the UK GPRD database. All lung cancer patients who have at least one record with one of the GPRD up-to-standard (UTS) practices, and who died during the study period (from 1 January 2000 to 31 December 2008) were included for analysis. The diagnosis of lung cancer was defined by Read/OXMIS codes. The Read/OXMIS codes used in this study are available upon request from authors. The UTS practices are those that contain at least 95% of all morbidity and prescribing information in the regular data audit (Walley and Mantgani, 1997). Pseudo-anonymised patients’ data on demographics, medical diagnoses, clinical consultations, analgesics prescription information, history of smoking and alcohol drinking status, referral and death information were recorded. Acquisition of access to the database for this study was funded by the UK Medical Research Council. The scientific and ethics advisory group of the GPRD approved the study. As anonymised electronic records were used as the source of data, written informed consent was not required.

### Outcome variable

1. The prescription prevalence, defined as the proportion of patients who received a certain category of analgesics (according to the WHO three-step analgesic ladder) during a defined time period compared with all patients with lung cancer during the same time period;
2. The binary indicator for whether a patient receives (status=1) analgesic prescription or not (status=0).

We investigated the prescription prevalence and binary status during the following three periods: last 1 to 3 months, last 3 to 6 months, and last 6 to 12 months. We examined level 1 (BNF code: 0407010; drugs including aspirin, paracetamol, non-steroidal anti-inflammatory analgesics, NSAIDs), level 2 (BNF code: 0407020; drugs including codeine, tramadol, and dihydrocodeine), and level 3 analgesics (BNF code: 0407020; drugs including morphine, diamorphine, fentanyl, and alfentanyl, buprenorphine, oxycodone, hydromorphone, methadone, pethidine, and meperidine). If patients received prescriptions of several levels of analgesics at the same time, they were counted at the highest level.

### Explanatory variables

Explanatory variables included age at diagnosis, gender, smoking status, year of death, social economic status, and regions. The first five were individual-level variables and the last two were practice-level variables. Age at diagnosis was the difference between the year of the first definite diagnostic code of lung cancer and the patient's year of birth. Age was grouped into seven groups (<30, 30–39, 40–49, 50–59, 60–69, 70–79, 80–89, 90+ years). Smoking status was recorded in the GPRD database as ‘current smoker’, ‘ex-smoker or unknown’, and ‘never smoker’. We classified a person as a ‘current smoker’ if their smoking status remained ‘current smoker’ across his/her whole consultation history. Similarly, ‘never smoker’ was the one whose smoking status remained being ‘never smoked’. All others or no consultation record of smoking status were categorised as ‘ex-smoker or unknown’. The date of death was determined using the procedure recommended by the GPRD. Year of death were in 1-year intervals from 2000 to 2008. The social economic status (SES) is a practice-level variable. It is calculated using the Index of Multiple Deprivation (IMD). It was expressed using the quintile with the lowest rank (0), indicating the least deprived, and the highest rank (4), indicating the most deprived. The region in the GPRD database was the NHS region in which the practice is based.

### Statistical analysis

The prescription prevalence and their 95% confidence intervals (95% CIs) were estimated. 95% CIs were constructed using normal approximation methods. The *χ*^2^-test was used to test whether proportions were significantly different between groups. The trends for proportions of patients receiving analgesics were estimated by the year of death, the age group, and the number of weeks before death.

To investigate what factors associate with whether a patient received a particular level of analgesics, we fitted the data with the log-binomial regression model. The dependent variable was a binary indicator of being prescribed a certain group of analgesics. Correlations within general practices (GP) were accounted for by using the generalised estimation equation method (Zeger *et al*, 1988). The working correlation matrix was specified as the exchangeable type. Adjusted proportion ratios (PRs) and their 95% CIs were estimated from the log-binomial models. The adjusted variables included age at diagnosis, gender, smoking status, year of death, SES, and region. We tested both main effects and interactions between independent factors. Since the GPRD database records only prescriptions that patients received from the general practitioners, it may miss prescriptions through non-GP routes. Therefore, we conducted a sensitivity analysis by repeating the main analysis using 3079 patients without referral records to any inpatient services within the last 18 months of life, to assess how the results might be affected by missed prescriptions.

All tests of statistical significance were two sided. We conducted analyses using SAS statistical software, version 9.1.3 (SAS Institute, Inc., Cary, NC, USA).

## Results

In total 10 202 patients died of lung cancer during the study period. Table 1 presents demographics of this population. 58.9% of the patients were diagnosed after the age of 70. The ratio of female-to-male lung cancer patients in this sample was 1 : 1.6 (39 *vs* 61%). Around one in three (29.3%) were current smokers and 13.0% were recorded as having never smoked. In the study sample, 28.2% (*n*=2880) of the patients were from GP in deprived areas (IMD score=4). The study population was widely spread across the UK.

In the last 3 months, overall prescription prevalence of the three levels of analgesics were 50.4% (95% CI: 49.4–51.4%) for level 1, 34.1% (95% CI: 33.2–35.0%) for level 2, and 55.5% (95% CI: 54.5–56.4%) for level 3. Prescription prevalences were significantly different by age, year of death, and region (*P*<0.05). But the differences were not significant for the comparisons between those with higher or lower social economic status for level 3 analgesics (*P*=0.39).

**Figure 1:**
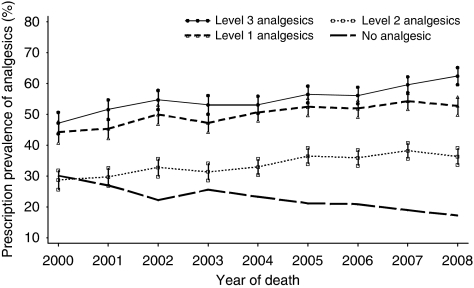
Time trend of prescription prevalence (95% CI) of level 1, level 2, and level 3 analgesics (according to the WHO analgesic ladder) in patients with lung cancer and proportion of patients without analgesic prescriptions during the last 3 months of life, 2000–2008 (*n*=10 202). Annual change: level 1: 1.2% (95% CI: 0.8–1.6%, *P*=0.001); level 2: 1.1% (95% CI: 0.8–1.5%, *P*<0.001); level 3: 1.5% (95% CI: 1.1–2.0%, *P*<0.0001); No prescription: −1.4% (95% CI: −1.8 to −1.0% *P*<0.001).

Prescription prevalence of any levels of analgesics in the last 3 months showed a small but significant increasing trend over the years (Figure 1). The annual increases were, respectively, 1.2% (95% CI: 0.8–1.6%, *P*=0.001), 1.1% (95% CI: 0.8–1.5%, *P*<0.001), and 1.5% (95% CI: 1.1–2.0%, *P*=0.0002) for level 1, level 2, and level 3 drugs. Level 3 analgesics prescription increased from 47.2% (95% CI: 43.7–50.7%) in 2000 to 62.4% (95% CI: 59.7–65.2%) in 2008. Over the period 2000–2008, around 50% of patients received level 1 or level 3 analgesic prescriptions, <40% of the patients had been prescribed level 2 analgesics. Prescriptions of level 3 analgesics were slightly lower than those of level 1, but experienced a dip from 2003 to 2004 and then sharper increase since 2005.

**Figure 2:**
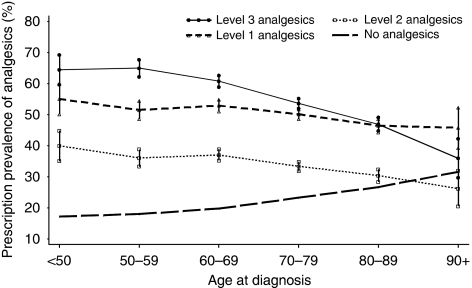
Prescription prevalence (95% CI) of level 1, level 2, and level 3 analgesics (according to the WHO analgesic ladder) in patients with lung cancer and proportion of patients without analgesic prescriptions by age groups (*n*=10 202). Average change per age group: level 1: −1.8% (95% CI: −2.5 to −1.2%, *P*=0.005); level 2: −2.6% (95% CI: −3.2 to −1.9%, *P*=0.002); level 3: −5.8% (95% CI: −7.6 to −4.1%, *P*=0.003); No prescription: 2.9% (95% CI: 2.2–3.6% *P*=0.001).

Prescription prevalence for all three levels of analgesics exhibited a linear and significant decreasing trend with increasing age (level 1: −1.8% (95% CI: −2.5 to −1.2%, *P*=0.005); level 2: −2.6% (95% CI: −3.2 to −1.9%, *P*=0.002); level 3: −5.8% (95% CI: −7.6 to −4.1%, *P*=0.003) (Figure 2). Analgesic prescriptions of level 1 (range: 45.8–55.1%) and level 3 (range: 36.0–65.0%) were both higher in all age groups than level 2 (range: 26.2–39.9%). Prescription prevalence of level 3 drugs were higher than those of level 1 in younger age groups (<70 years) but started dropping from the ‘70–79’ age group to lower than level 1 in the ‘90+’ group.

People without any analgesics prescriptions in the last 3 months presented a downward trend by year of death (−1.4% 95% CI: −1.8 to −1.0% *P*<0.001) and an upward trend by age group (2.9% 95% CI: 2.2–3.6% *P*=0.001), the mirror-reflection images of prescription prevalence of analgesics, particularly for level 3 analgesics (Figures 1 and 2).

Log-binomial regression analysis (Table 2) showed that age is a significant predictor for a patient receiving analgesics prescriptions in the last 3 months of life. The chance of a patient being prescribed analgesics was negatively associated with age. The chance starts dropping from the ‘70–79’ age group; the lowest level is in the ‘90+’ group. Patients diagnosed after the age of 90 years are less likely to receive a level 3 (PR: 0.55; 95% CI: 0.45–0.67), level 2 (PR: 0.66; 95% CI: 0.52–0.85), or level 1 (PR: 0.80; 95% CI: 0.67–0.96) analgesic prescription than those diagnosed before the age of 50 years, respectively. Females had a slightly higher chance to receive level 3 (PR: 1.06; 95% CI: 1.02–1.10) analgesics than males. There was no gender difference in level 1 (PR: 0.99; 95% CI: 0.95–1.02) or level 2 analgesic prescriptions (PR: 1.05; 95% CI: 0.99–1.10).

Never-smokers were less likely than smokers to be prescribed level 2 analgesics (PR: 0.90; 95% CI: 0.82–1.00), but no difference in receiving the other levels of analgesic prescriptions. After adjusting for the effects of all other predictors, the annual increasing trend was still significant for all levels of analgesics. Patients registered with practices in least deprived regions had a slightly lower chance (*P*>0.05) of receiving any levels of analgesics than in most deprived regions in the last 3 months, with the PRs ranging from 0.93 to 0.99. There are significant regional differences in prescribing analgesics. Patients in London had the least chance to get a prescription of any level of analgesics. Those in the Southern region had the highest chance of being prescribed level 3 analgesics. We did not find significant interaction effects between independent variables.

Results of sensitivity analysis (Appendix Table A1) were generally consistent with our main findings but fewer factors reached a significant level, probably due to smaller sample size.

## Discussion

Our analysis showed an increasing trend of prescribing level 3 analgesics (strong opioids) in UK primary care. This is consistent with a Norwegian study using a national prescription database (Fredheim *et al*, 2010). In the last 3 months, patients receiving level 3 analgesic prescriptions increased from one in two (47.2%) in 2000 to two in three (62.4%) in 2008. By 2008, two in three patients had been prescribed in the last 3 months of life level 1 (52.8%) and level 3 (62.4%) analgesics, and nearly one in two level 2 analgesics (36.4%). The increasing prescribing trend of analgesics towards end of life was similar to that found in a smaller Dutch survey of general practitioners (Borgsteede *et al*, 2009). This suggests that this pattern may well be replicated in other countries.

Interestingly, between 2003 and 2004 there was a small dip in analgesic prescribing in primary care. At around this time, the case of Harold Shipman (the doctor who murdered patients using opioids) was dramatised and made known to professionals and the wider public. There have been some concerns that this case would adversely affect appropriate opioid prescribing and pain management (Baker *et al*, 2004). We find a small amount of evidence to support a small dip in prescribing – the effect was short term though, and the upward trend in opioid prescribing continued. However, this finding highlights the need for continued education in opioids, and the prevalent use in the last 3 months of life suggests that community services need to be trained in the administration, including out of hours. This may be important in ensuring support at home (Gomes and Higginson, 2006).

However, we found older age being significantly and independently associated with lower prescriptions of any levels of analgesics in primary care. Though analgesic prescribing is a balance of benefits *vs* harms, and this balance can sometimes be difficult to achieve, mounting evidence in the other settings consistently suggested pain management is more problematic for older people (Cleeland *et al*, 1994; Bernabei *et al*, 1998; Closs *et al*, 2009; Oldenmenger *et al*, 2009). Reasons why older people are apparently undertreated for pain are likely to be multifaceted: (1) it may be that older people are less likely to report pain; (2) they may have atypical manifestations of pain; (3) there may be patients’ misconceptions about tolerance and addiction to opioids; (4) there may be co-morbidities that limit treatments; or (5) the needs of older people with lung cancer may be missed (Oldenmenger *et al*, 2009). It is known that specialist palliative care services have seemed to focus on younger patients, rather than those in older age groups (Eve and Higginson, 2000; Lock and Higginson, 2005) and much of the opioid prescriptions may be prompted by palliative care teams. Therefore, it is likely that older people with lung cancer and pain are not receiving optimal treatment. Adequate assessment of pain is key to effective management (Oldenmenger *et al*, 2009), but this will require better integration of pain assessment into busy clinical practices.

Gender differences in pain and analgesia have been increasingly reported in basic research as well as in clinical settings: women are more sensitive to pain but less responsive to analgesic treatment than men (Fillingim and Gear, 2004; Potter and Higginson, 2004; Gaumond *et al*, 2007). This may explain why female patients in our study exhibited a slightly higher chance of receiving analgesic prescriptions in the last year of life. The mechanisms behind the phenomenon may be attributed to multiple factors, from genes and reproductive hormones to socio-cultural and environmental factors (Mogil, 1999; Craft *et al*, 2004; Greenspan *et al*, 2007; Nielsen *et al*, 2008), all may play a part.

Smoking is a well-recognised risk factor for lung cancer, accounting for around 85–90% of cases (Cancer Research UK, 2009). An American study found that persistent smoking after a diagnosis of lung cancer is associated with higher pain levels (Daniel *et al*, 2009). Another study of 112 newly diagnosed head and neck cancer patients also observed that current smokers reported higher general and oral pain levels than did never and former smokers (Logan *et al*, 2009). Our findings do not support these observations – in our study only level 2 analgesics were slightly lower in non-smokers (30.0 *vs* 35.1%). The main difference between our study and the earlier work is that we focused on the last 3 months of a patient's life. Whether the effects of smoking on pain wane at the end of life and subsequently influence the chance of a patient getting analgesic prescriptions would need further study.

A review of published data found socio-economical variables being the strongest determinant for undertreatment (Deandrea *et al*, 2008). Our results suggest a tendency for patients from deprived backgrounds receiving more analgesic prescriptions from their GPs. However, the difference is not statistically significant. We are not able to exclude the possibility of undertreatment of pain, but the reimbursement policy in the United Kingdom may be of help in reducing any inequality in pain relief (Cherny *et al*, 2010).

This study revealed an interesting finding in regional differences in analgesics prescriptions. Despite London having a relatively high density of palliative services (as do the South East and North West regions), prescribing rates are low. A possible reason might be the imbalance in service provision. A national survey for palliative care service provision across the United Kingdom found that London has the lowest level of day care places, though the other service categories rank high (National Council for Hospice and Specialist Palliative Care Services, 2000). Scientific evidence suggests that palliative day care service is associated with improved pain and symptom control (Goodwin *et al*, 2003); our results also highlight the importance of enhancing palliative day care service.

Several limitations of our study need to be recognised. First, since the GPRD database only record analgesics prescribed by GPs, we may miss some analgesic prescriptions that patients received through non-GP routes; therefore, the prescriptions reported here were generally underestimated. This is particularly true for level 1 and level 2 analgesics, which patients can buy directly at a pharmacy without a prescription. It may have been used especially by patients from higher socio-economic backgrounds. Equally, some level 3 analgesics may have been provided by hospitals, and we do not know what analgesics were prescribed once patients entered hospitals or hospices, which may be the likely situation during the last several weeks of a patient's life. Furthermore, prescriptions are normally written for a 3-month duration, so in the later months patients may be using their earlier drugs (possibly at increased dose) without needing another prescription. The issue is more of a problem in the later part of the end of life journey than in the early. However, the sensitivity analysis showed such underestimation does not have major impact on our main results.

Second, restricted by data availability, we could not evaluate analgesic prescriptions in relation to actual pain prevalence and intensity. Pain was under-recorded in primary care. In the last 3 months, only 16.3% (1668 out of 10 202) of the patients had pain recorded in their clinical histories, in contrast to an overall 55.5% of patients having been prescribed level 3 analgesics. Effective cancer pain management relies heavily on accurate assessment of the nature and severity of the patient's pain. We did not have this information since pain assessment is not routinely performed and recorded in most settings including primary care (Oldenmenger *et al*, 2009); however, we would like to emphasise the need for education and training of clinicians on the value of documenting pain assessments, on appropriate use of medications guided by the WHO analgesic principle, on monitoring any adverse effects and on maintaining communication with patients and families (Bruera and Kim, 2003).

Third, the factors we could adjust for in multiple regression analysis were limited. For example, ‘ethnic group’ may be one of the influencing factors for optimal pain management, but we do not have this information. Finally, it should be noted that some prescriptions at level 3 would include level 2 or level 1 drugs. However, this does not invalidate our findings, as it would represent appropriate treatment.

In conclusion, we found an increasing trend in UK general practitioners prescribing all levels of analgesics in the last 3 months of lung cancer patients. However, people older than 70 years of age were relatively under-prescribed for all levels of analgesics, particularly at level 3; therefore, warranting further investigation and suggesting attention to the needs of older lung cancer patients.

## Figures and Tables

**Table 1:** Demographics of lung cancer patients who died between 1 January 2000 and 31 December 2008 in the General Practice Research Database (GPRD)

**Variable**	***N* (%)**
All groups	10 202
*Age at diagnosis (years)*	
<50	383 (3.8)
50–59	1145 (11.2)
60–69	2661 (26.1)
70–79	3759 (36.9)
80–89	2029 (19.9)
90+	225 (2.2)
*Gender*	
Female	3982 (39.0)
Male	6220 (61.0)
*Smoking*	
Never	1325 (13.0)
Current	2992 (29.3)
Ex-smoker or unknown	5886 (57.7)
*Year of death*	
2000	790 (7.7)
2001	952 (9.3)
2002	998 (9.8)
2003	1045 (10.2)
2004	1229 (12.1)
2005	1290 (12.6)
2006	1321 (13.0)
2007	1400 (13.7)
2008	1177 (11.5)
*Social-economic status (SES)*	
0 (Least deprived)	1747 (17.1)
1	1720 (16.9)
2	1871 (18.3)
3	1984 (19.5)
4 (Most deprived)	2880 (28.2)
*Region*	
Eastern	1335 (13.1)
London	959 (9.4)
North East	897 (8.8)
North West and West Midland	2688 (26.4)
Northern Ireland	282 (2.8)
Scotland	715 (7.0)
Southern	2692 (26.4)
Wales	634 (6.2)

**Table 2:** Prevalence ratios (PRs)^a^ of factors associated with a patient receiving three levels of analgesic prescriptions during the last 3 months of life (*n*=10 202)

	**Level 1**		**Level 2**		**Level 3**	
**Variable**	**PR (95% CI)**	***P***	**PR (95% CI)**	***P***	**PR (95% CI)**	***P***
*Age (years)*						
<50	1.00	<0.001	1.00	<0.001	1.00	<0.001
50–59	0.93 (0.83–1.03)		0.89 (0.77–1.03)		1.02 (0.93–1.10)	
60–69	0.94 (0.85–1.04)		0.90 (0.79–1.03)		0.94 (0.87–1.02)	
70–79	0.89 (0.81–0.98)		0.82 (0.72–0.93)		0.84 (0.77–0.90)	
80–89	0.82 (0.73–0.91)		0.74 (0.65–0.85)		0.73 (0.66–0.79)	
90+	0.80 (0.67–0.96)		0.66 (0.52–0.85)		0.55 (0.45–0.67)	
*Gender*						
Male	1.00	0.46	1.00	0.09	1.00	0.002
Female	0.99 (0.95–1.02)		1.05 (0.99–1.10)		1.06 (1.02–1.10)	
*Smoking status*						
Smoker	1.00	0.006	1.00	0.12	1.00	0.27
Ex-smoker or unknown	1.07 (1.03–1.12)		0.99 (0.93–1.05)		1.03 (0.99–1.07)	
Never smoker	1.05 (0.98–1.13)		0.90 (0.82–1.00)		0.99 (0.93–1.05)	
*Year of death*						
2000	1.00	<0.001	1.00	<0.001	1.00	<0.001
2001	1.03 (0.92–1.15)		1.03 (0.89–1.21)		1.10 (0.99–1.21)	
2002	1.14 (1.03–1.25)		1.14 (0.98–1.32)		1.19 (1.08–1.31)	
2003	1.08 (0.97–1.20)		1.10 (0.95–1.26)		1.15 (1.05–1.27)	
2004	1.15 (1.04–1.26)		1.14 (1.00–1.31)		1.14 (1.03–1.26)	
2005	1.19 (1.08–1.31)		1.27 (1.11–1.45)		1.22 (1.11–1.34)	
2006	1.18 (1.07–1.29)		1.25 (1.09–1.43)		1.20 (1.09–1.32)	
2007	1.22 (1.12–1.34)		1.32 (1.17–1.50)		1.27 (1.15–1.40)	
2008	1.19 (1.08–1.31)		1.26 (1.10–1.43)		1.34 (1.22–1.47)	
*Socio-economic status (SES)*						
0 (Least deprived)	1.00	0.31	1.00	0.35	1.00	0.27
1	0.97 (0.90–1.06)		1.04 (0.94–1.14)		0.99 (0.93–1.07)	
2	1.03 (0.96–1.11)		1.06 (0.95–1.18)		1.02 (0.95–1.09)	
3	1.03 (0.96–1.10)		0.99 (0.90–1.10)		1.04 (0.97–1.12)	
4 (Most deprived)	1.05 (0.98–1.12)		1.08 (0.98–1.19)		1.06 (0.99–1.13)	
*Region*						
Wales	1.00	0.38	1.00	<0.001	1.00	0.002
Eastern	0.99 (0.90–1.10)		0.87 (0.77–0.99)		1.06 (0.95–1.18)	
London	0.99 (0.90–1.10)		0.87 (0.77–0.99)		1.06 (0.95–1.18)	
North East	0.96 (0.86–1.08)		0.79 (0.69–0.90)		1.01 (0.90–1.14)	
North West and West Midlands	1.06 (0.95–1.18)		0.99 (0.86–1.14)		1.13 (1.01–1.27)	
Northern Ireland	1.04 (0.94–1.14)		0.95 (0.85–1.08)		1.10 (0.99–1.21)	
Scotland	1.03 (0.90–1.17)		1.18 (1.02–1.37)		1.12 (0.97–1.31)	
Southern	1.06 (0.95–1.19)		1.01 (0.87–1.18)		1.14 (1.02–1.28)	

Abbreviation: CI=confidence interval.

a PRs were estimated using log-binomial regression model, the listed variables in the table were including in the model as the independent variables.

**Table A1:** Prevalence ratios (PRs)^a^ of factors associated with a patient receiving three levels of analgesic prescriptions during the last 3 months of life, based on patients without referral records to any of inpatient services (*n*=3072)

	**Level 1**		**Level 2**		**Level 3**	
**Variable**	**PR (95% CI)**	***P***	**PR (95% CI)**	***P***	**PR (95% CI)**	***P***
*Age (years)*						
<50	1.00	0.010	1.00	0.002	1.00	<0.001
50–59	0.86 (0.72–1.02)		0.89 (0.69–1.16)		0.96 (0.83–1.11)	
60–69	0.86 (0.74–1.00)		0.95 (0.75–1.20)		0.90 (0.78–1.03)	
70–79	0.84 (0.72–0.97)		0.79 (0.62–0.99)		0.83 (0.73–0.94)	
80–89	0.72 (0.61–0.85)		0.73 (0.57–0.93)		0.68 (0.58–0.80)	
90+	0.80 (0.61–1.04)		0.66 (0.43–1.03)		0.61 (0.43–0.85)	
*Gender*						
Male	1.00	0.22	1.00	0.53	1.00	0.74
Female	0.96 (0.89–1.03)		1.03 (0.94–1.13)		1.01 (0.95–1.07)	
*Smoking status*						
Smoker	1.00	0.66	1.00	0.12	1.00	0.41
Ex-smoker or unknown	1.03 (0.95–1.11)		0.98 (0.88–1.09)		0.95 (0.89–1.02)	
Never smoker	1.05 (0.93–1.18)		0.83 (0.68–1.01)		0.96 (0.87–1.07)	
*Year of death*						
2000	1.00	0.035	1.00	0.13	1.00	0.022
2001	1.02 (0.82–1.27)		1.00 (0.76–1.31)		0.97 (0.81–1.16)	
2002	1.06 (0.89–1.27)		1.03 (0.78–1.37)		1.06 (0.91–1.23)	
2003	1.05 (0.86–1.29)		0.92 (0.71–1.20)		0.98 (0.83–1.16)	
2004	1.10 (0.92–1.33)		1.02 (0.79–1.31)		1.00 (0.85–1.19)	
2005	1.25 (1.04–1.51)		1.15 (0.89–1.48)		1.10 (0.93–1.30)	
2006	1.17 (0.97–1.40)		1.18 (0.92–1.51)		1.08 (0.92–1.26)	
2007	1.17 (0.97–1.40)		1.21 (0.95–1.53)		1.12 (0.96–1.31)	
2008	1.24 (1.04–1.47)		1.19 (0.95–1.49)		1.19 (1.02–1.39)	
*Socio-economic status (SES)*						
0 (Least deprived)	1.00	0.91	1.00	0.68	1.00	0.58
1	0.97 (0.86–1.10)		0.99 (0.85–1.15)		0.95 (0.85–1.05)	
2	1.03 (0.92–1.15)		0.97 (0.82–1.16)		0.98 (0.90–1.07)	
3	0.98 (0.88–1.09)		0.89 (0.75–1.06)		1.00 (0.91–1.09)	
4 (Most deprived)	1.02 (0.91–1.13)		1.00 (0.84–1.17)		1.04 (0.95–1.13)	
*Region*						
Wales	1.00	0.10	1.00	0.19	1.00	0.020
Eastern	1.04 (0.86–1.24)		0.91 (0.75–1.12)		1.10 (0.94–1.28)	
London	1.17 (0.97–1.41)		0.86 (0.69–1.07)		1.13 (0.98–1.30)	
North East	1.12 (0.93–1.35)		1.10 (0.87–1.38)		1.33 (1.14–1.56)	
North West and West Midlands	1.08 (0.91–1.27)		0.95 (0.78–1.15)		1.22 (1.07–1.39)	
Northern Ireland	0.96 (0.74–1.23)		1.10 (0.79–1.53)		1.18 (0.98–1.41)	
Scotland	1.17 (0.96–1.42)		1.00 (0.76–1.32)		1.28 (1.10–1.50)	
Southern	0.98 (0.83–1.16)		0.84 (0.69–1.02)		1.23 (1.08–1.41)	

Abbreviation: CI=confidence interval.

a PRs were estimated using log-binomial regression model, the listed variables in the table were including in the model as the independent variables.

## APPENDIX

Table A1
